# Elevated high-sensitivity C-reactive protein combined with procalcitonin predicts high risk of contrast-induced nephropathy after percutaneous coronary intervention

**DOI:** 10.1186/s12872-019-1137-9

**Published:** 2019-06-24

**Authors:** Guoqiang Gu, Xuechao Yuan, Yaqing Zhou, Demin Liu, Wei Cui

**Affiliations:** Department of Cardiology, Second Hospital of Hebei Medical University, No. 215 Hepingxi Road, Shijiazhuang, 050000 Hebei China

**Keywords:** Contrast-induced nephropathy, High-sensitivity C-reactive protein, Hydration, Procalcitonin, Percutaneous coronary intervention

## Abstract

**Background:**

Contrast-induced nephropathy (CIN) is common after percutaneous coronary intervention (PCI) and always leads to a poor prognosis. Compared with conventional detection methods, either high-sensitivity C-reactive protein (hs-CRP) or procalcitonin have higher sensitivity and specificity for predicting CIN, but their combination has not been explored. This prospective study investigated the value of hs-CRP combined with procalcitonin for predicting CIN after PCI.

**Methods:**

All patients undergoing PCI admitted to our hospital during the year 2016 were consecutively enrolled (*n* = 343). The patients received adequate hydration before PCI and 20 mg furosemide after the procedure. CIN was diagnosed by a 25% elevation in serum creatinine or ≥ 44.2 μmol/L (0.5 mg/dL) serum creatinine within 48 to 72 h after intravenous injection of contrast media.

**Results:**

Patients with high hs-CRP or procalcitonin had higher rates of CIN relative to those patients with low values. For predicting CIN, hs-CRP combined with procalcitonin showed an area under the receiver operating characteristic curve of 0.67, with optimal cut-off value 0.0643610, and the sensitivity and specificity were higher than hs-CRP or procalcitonin alone. The logistic regression analysis showed that high-risk factors of CIN were acute myocardial infarction and highly elevated hsCRP and procalcitonin.

**Conclusions:**

Prior to PCI, an elevation of the inflammatory biomarkers hsCRP and procalcitonin are a risk factor for postoperative CIN. This study suggests that the combination of hsCRP and procalcitonin is a better predictor of CIN after PCI then either hsCRP or procalcitonin alone.

**Trial registration number:**

ChiCTR-IOR-14005250. Date of registration 2014-09-24.

## Background

Contrast-induced nephropathy (CIN), also known as contrast-induced acute kidney injury, refers to acute kidney injury that occurs after the use of contrast media [1]. CIN is defined as 25% higher serum creatinine than before intravenous injection of contrast media, or 44.2 μmol/L (0.5 mg/dL) in absolute value within 48 to 72 h, with other causes of renal damage excluded. CIN is a common complication after percutaneous coronary intervention (PCI).

The application of PCI has resulted in a significant increase in the incidence of CIN. CIN now accounts for ~ 11% of iatrogenic acute kidney injuries, which is second only to drugs and renal ischemia-induced acute kidney injury [2, 3]. The high incidence of CIN is especially problematic in high-risk patients with coronary heart disease, diabetes mellitus, congestive heart failure, or chronic kidney disease [2]. Furthermore, the average hospital cost of patients with CIN is significantly higher than that of patients without CIN [4], and CIN dramatically increases rates of patients’ hospitalization and mortality long-term [5]. Therefore, early recognition of high-risk groups, such as those with diabetes, renal insufficiency, hypertension, and advanced age, is particularly crucial for active prevention and treatment of CIN. Although various predictive scoring systems for CIN based on risk factors have been developed [6, 7], novel biomarkers may also help identify high-risk patients.

C-reactive protein (CRP) is synthesized by the liver under stress and participates in inflammatory reactions. In healthy individuals or patients with stable disease, the concentration of CRP is stable [8]. Gao et al. [9] analyzed over 7000 PCIs and found that, relative to patients without CIN, those with CIN had significantly higher preoperative levels of CRP.

Procalcitonin (PCT) is a stable glycoprotein both in vitro and in vivo. Serum PCT in healthy individuals is extremely low (< 0.1 μg/L) [10]. PCT levels rise earlier than other inflammatory factors in response to severe infection, acute trauma, or other factors [11]. Kurtul et al. [12] found that elevated PCT was an independent risk factor for CIN in 814 patients with acute coronary syndrome undergoing PCI.

Although either CRP or PCT alone has little value for predicting the risk of CIN, their combined value has not been explored. Thus, this prospective study investigated the value of hs-CRP combined with procalcitonin for predicting CIN after PCI.

## Methods

The Ethics Committee of Second Hospital of Hebei Medical University approved this prospective study. All patients signed the written informed consent and participated voluntarily.

### Patients

All patients undergoing PCI admitted to our hospital from 1 January 2016 to 31 December 2016 were prospectively enrolled in this study. Patients with any of the following were excluded: allergic to contrast media; contrast media exposure within 2 days prior to PCI; emergency PCI; chronic kidney disease or post-transplantation; malignant tumor; autoimmune disease; recent surgery, or a history of trauma within 1 month; fever or infection within 1 month diagnosed according to the infective symptoms (salivation, cough), physical examination, laboratory tests, or chest X-ray or CT image; or acute or chronic inflammatory diseases.

The final study population comprised 343 patients (247 men and 96 women). Ages ranged from 27 to 77 years, with a mean age 57.86 ± 9.16 years. The average age of the men and women were 56.6 ± 9.3 years and 61.1 ± 7.9 years, respectively.

All patients received continuous hydration at 1 mL·kg^− 1^·h^− 1^ (about 2000 mL of normal saline), from 4 h before PCI to 24 h after PCI, and a dose of furosemide (20 mg) was given immediately after PCI.

For this analysis, the patients were compared as being with or without CIN after PCI (i.e., CIN or non-CIN). CIN was defined as an increase of preoperative serum creatinine by 25%, or total ≥ 44.2 μmol/L (0.5 mg/dL), within 48 to 72 h after intravenous injection of contrast media [13]. Serum creatinine was tested before and at 48 h after the procedure.

### Data collection

Patients’ baseline characteristics and medication histories were collected. Routine laboratory data before and after the PCI procedures included routine tests of blood, urine, and stool, liver and kidney function, serum lipids and glucose, brain natriuretic peptide, myocardial enzyme, CRP, and PCT. All the blood variables were measured using an autoanalyzer (Hitachi 747; Hitachi, Tokyo, Japan) at our central laboratory.

### Testing of CRP and PCT

Venous blood specimens were centrifuged at 200×*g* for 10 min, and serum was tested for high-sensitive CRP (hs-CRP) and PCT. The levels of hs-CRP were measured using a commercial, high-sensitivity nephelometric assay (Cias Latex CRP-H, Kanto Chemical, Tokyo, Japan). PCT was tested using a solid-phase sandwich enzyme-linked immunosorbent assay (PCT kit, SEA689Hu, Chinese and American Technology, China).

PCT and hs-CRP were each differentiated as low or high according to the median cutoffs, 97.47 pg/mL and 2.1 mg/L, respectively. Because CRP and PCT are biomarkers of inflammation, the patients were also stratified into low-, medium-, or high-inflammation groups based on their combined CRP and PCT status. Specifically, low inflammation was defined as low CRP and low PCT. Medium inflammation was considered the combination low CRP and high PCT, or high CRP and low PCT. In the high-inflammation group, both CRP and PCT were high.

### Statistical methods

All statistical analysis was performed with SPSS for Windows (version 18, SPSS, Chicago, IL, USA) and Medcalc software. For normally distributed data, continuous variables are shown as mean ± standard deviation. For comparisons between the CIN and non-CIN groups, the independent-sample *t*-test was used. Otherwise, the median and interquartile range (25–75%) values are displayed, and non-parametric tests were conducted. Categorical variables are shown as number and percentage (%) and compared using the chi-squared test. The software program MedCalc was utilized to measure the receiver operating curve (ROC) for determining the differences between the area under ROC curve (AUC) of each biomarker for distinguishing between CIN and non-CIN patients. Multiple logistic regression analysis was used to identify independent risk factors of CIN. A statistical difference was defined as *P* < 0.05.

## Results

### Baseline characteristics of the patients

The CIN and non-CIN groups (*n* = 28, 315, respectively) were comparable with regard to rates of medication history and underlying diseases, including hypertension, diabetes mellitus, and hyperlipidemia (Table 1). The average dose of contrast media in the CIN group was 1.6 times the dose of the non-CIN group. However, this difference did not reach a statistical difference. A significantly higher percentage of the CIN group had experienced acute myocardial infarction (AMI; 57.1%) compared with the non-CIN group (32.7%; *P* = 0.009). There were no significant differences between the CIN and non-CIN groups concerning the percentage of heart failure and ejection fraction. However, the mean left ventricular size of the CIN group was significantly larger than that of the non-CIN group (*P* = 0.014).

**Table 1:** Baseline characteristics and contrast media dosage of the CIN and non-CIN groups

		Non-CIN	CIN	*P*-value
Subjects, n		315	28	–
Males, n (%)		228 (72.4)	19 (67.9)	0.609
Age, y	< 45	24 (7.6)	1 (3.6)	0.723
	45–59	144 (45.7)	14 (50)	
	60–74	140 (44.5)	13 (46.4)	
	≥75	7 (2.2)	0 (0.0)	
Weight, kg		71.0 (65.0–80.0)	70.0 (65.0–78.0)	0.643
Height, m		1.7 (1.6–1.7)	1.7 (1.6–1.7)	0.571
Smoking		136 (43.2)	10 (35.7)	0.444
Underlying diseases	Hypertension	188 (59.7)	19 (67.9)	0.397
	Diabetes mellitus	79 (25.1)	5 (17.9)	0.394
	Hyperlipidemia	144 (45.7)	10 (35.7)	0.308
Medication history	ACEI or ARB	138 (43.8)	16 (57.1)	0.174
	Statins	290 (92.1)	24 (85.7)	0.277
	AMI	103 (32.7)	16 (57.1)	0.009
Before PCI	Multi-vessel lesions	85 (27.0)	9 (32.1)	0.558
	Heart failure	66 (21.0)	9 (32.1)	0.170
	Ejection fraction	61.8 (60.6–63.1)	61.3 (57.7–63.6)	0.303
	LV, mm	47.0 (45.0–50.0)	49.5 (46.3–52.0)	0.014
Types of CM	Iso-osmotic	11 (3.5)	4 (14.3)	0.026
	Low-osmotic	304 (96.5)	24 (85.7)	
Contrast dose, mL		100 (100–160)	100 (100–160)	0.327
Hemoglobin, g/L		138.2 ± 12.7	139.0 ± 11.4	0.748
SCr, μmol/L		69.3 (60.1–77.4)	60.4 (51.5–64.7)	0.000
BUN, mmol/L		5.1 (4.3–6.1)	5.1 (3.9–5.7)	0.549
Cystatin C, mg/L		1.0 (0.9–1.1)	1.0 (0.9–1.1)	0.981
FBG, mmol/L		4.9 (4.4–5.8)	5.6 (4.8–6.7)	0.016
TC, mmol/L		4.2 (3.6–4.9)	4.5 (3.9–4.8)	0.328
Homocysteine, μmol/L		16.2 (13.3–22.0)	17.2 (13.1–22.8)	0.660
Albumin, g/L		41.3 ± 3.5	40.5 ± 3.8	0.259
Contrast media, mL		100 (100–160)	160 (100–160)	0.327

Abbreviations: *ACEI* Angiotensin-converting enzyme inhibitors, *AMI* Acute myocardial infarction, *ARB* Aangiotensin receptor blocker, *BUN* Blood urea nitrogen, *EF* Ejection fraction, *FBG* Fasting blood glucose, *LV* Left ventricle, *SCr* Serum creatinine, *TC*, Total cholesterol

Four patients in the CIN group (14.3%) and 11 in the non-CIN group (3.5%) received iodixanol (*P* = 0.026), but there were no significant differences in the doses of contrast media between the 2 groups (Table 1). The 2 groups were also statistically similar with regard to pre-PCI levels of hemoglobin, blood urea nitrogen, cystatin C, fasting blood glucose, total cholesterol, homocysteine, and albumin.

### Incidence rates of CIN in different subgroups according to hs-CRP and PCT

The median hs-CRP and PCT in enrolled patients were 2.1 mg/L and 97.47 pg/mL, respectively. The patients were further stratified as preoperative low or high hs-CRP (< 2.1 or ≥ 2.1 mg/L, respectively) and low or high PCT (PCR < 97.47 or ≥ 97.47 pg/mL; Table 2). The rates of CIN were higher in the high hs-CRP or PCT groups relative to the low groups.

**Table 2:** Incidence rates of CIN varied according to low or high hs-CRP and PCT *

		Non-CIN	CIN	*P*
Subjects, n		315	28	
Hs-CRP	Low	157 (49.8)	8 (28.6)	0.031
	High	158 (50.2)	20 (71.4)	
PCT	Low	163 (51.7)	8 (28.6)	0.019
	High	152 (48.3)	20 (71.4)	
Inflammation	Low	86 (98.9)	1 (1.1)	0.008
	Medium	148 (91.4)	14 (8.6)	
	High	81 (86.2)	13 (13.8)	

* Low hs-CRP is considered hs-CRP < 2.1 mg/L; high hs-CRP is ≥2.1 mg/L. Low PCT is defined as PCR < 97.47 pg/mL; high PCR is ≥97.47 pg/mL

Because CRP and PCT are biomarkers of inflammation, the patients were also stratified into low-, medium-, or high-inflammation groups. The incidence rates of CIN rose with the inflammation status (*P* = 0.008).

### Value of hs-CRP and PCT in predicting CIN by ROC

**Fig. 1:**
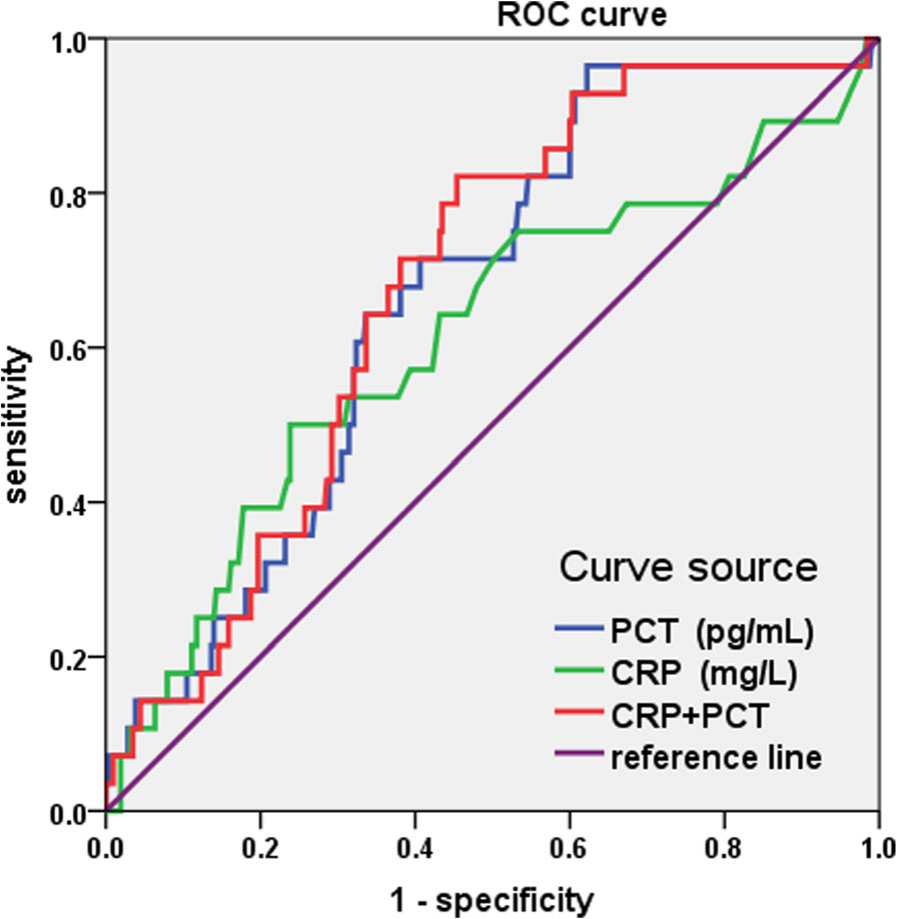
ROC curves for predicting CIN

The values of hs-CRP and PCT in predicting CIN were analyzed by constructing receiver operating characteristic (ROC) curves (Table 3; Fig. 1). Regarding hs-CRP, the area under the ROC curve (AUC) for predicting CIN was 0.612. The optimal cut-off value was 4.750 mg/L with a sensitivity of 50.0% and specificity of 76.2% (95% confidence interval [CI] 0.493–0.731; *P* = 0.050). For PCT, the AUC was 0.666, and the optimal cut-off value was 65.945 pg/mL with a sensitivity of 96.4% and specificity of 37.8% (95% CI: 0.575–0.757; *P* = 0.004).

**Table 3:** Predictive values of hs-CRP and PCT for CIN by ROC curves

	AUC	*P*	95% CI	Sensitivity	Specificity	Cut-off value
Hs-CRP	0.612	0.050	0.493–0.731	50.0%	76.2%	4.750 mg/L
PCT	0.666	0.004	0.575–0.757	96.4%	37.8%	65.945 pg/mL
Hs-CRP + PCT	0.678	0.002	0.590–0.767	82.1%	54.6%	0.0643610

When combining hs-CRP and PCT, the AUC was 0.67. The optimal cut-off value was 0.0643610, with a diagnostic sensitivity of 82.1% and specificity 54.6% (95% CI: 0.590–0.767; *P* = 0.002).

### Multiple logistic regression analysis of risk factors for CIN

The multiple logistic regression analysis included the following factors: AMI; multivessel lesions; contrast media type; contrast media dosage; left ventricular size; fasting blood glucose; inflammatory biomarkers; and traditional risk factors such as hypertension, diabetes, age, heart failure, and anemia.

Results of the multiple logistic regression showed that both AMI and inflammatory biomarkers were high-risk factors of CIN (Table 4). The incidence of CIN was higher in patients in whom both hs-CRP and PCT were elevated (the high inflammation group), compared with elevations of either hs-CRP or PCT alone (low or medium inflammation). The OR value of CIN was 2.352 with 95.0% CI 1.279–4.325.

**Table 4:** Multiple logistic regression analysis of risk factors for CIN

		B	S.E.	Wald	Sig.	Exp(B)	95.0% CI for Exp(B)
Inflammation	Low	Reference					
	High PCT or Hs-CRP	2.305	1.066	4.678	0.031	10.022	1.241–80.905
	High PCT + Hs-CRP	2.709	1.068	6.432	0.011	15.017	1.850–121.868
FBG		0.245	0.114	4.63	0.031	1.278	1.022–1.597

## Discussion

In this study, we investigated whether the combined values of the inflammatory biomarkers hs-CRP and PCT may predict the development of CIN after PCI. Among 343 patients who underwent PCI at our hospital during the year 2016, 28 (8.16%) subsequently experienced CIN. The study determined that patients with high preoperative levels of either hs-CRP or PCT were more likely to develop CIN, compared with patients with lower values. Notably, high preoperative levels of both hs-CRP and PCT were significantly more predictive of CIN, relative to that of lower values of either. The multiple logistic regression analysis confirmed that elevated preoperative levels of hs-CRP together with PCT were a risk factor for CIN after PCI. The study also found that AMI was an independent risk factor for CIN. According to these results, the combination of hs-CRP and PCT can be used to predict an increased risk of CIN after PCI, and is more accurate than either biomarker alone.

Most previous studies [12, 14] have focused on the efficacy of using either hs-CRP or PCT to predict post-PCI CIN. The predictive value of high hs-CRP has been confirmed [9, 14]. Kurtul et al. [12] analyzed 814 patients with acute coronary syndrome undergoing PCI, and determined that elevated PCT increased the risk of CIN. However, few studies have explored the value of combining hs-CRP and PCT to predict CIN. In the present study, we found that the combination of these 2 biomarkers had a higher predictive value for CIN than did either by itself. Multiple logistic regression analysis revealed that the risk of CIN among patients with elevated Hs-CRP and PCT was 2.35-fold higher than for those with elevated Hs-CRP or PCT alone. Therefore, the value of combining hs-CRP and PCT before PCI for predicting the risk of postoperative CIN was confirmed. This combined indicator could signal the need for early preventive measures, efficiently prevent CIN or improve its prognosis, and finally reduce the associated medical burden.

The mechanisms by which elevated hs-CRP and PCT promote the development of CIN is not fully understood. It has been reported that the onset of CIN may be related to the production of inflammatory cytokines and chemokines in the kidney, the upregulation of leukocyte adhesion factor, and the infiltration of various inflammatory cells into the kidney [15–17]. It may be that CRP and PCT not only serve as indicators of inflammation, but are also involved in the inflammatory response and thus increase the risk of CIN.

In addition, CIN after PCI may be related to reactive oxygen species-induced oxidative stress. In a recent report, Raddant et al. [18] concluded that reactive oxygen species were associated with PCT levels. Contrast media affects the apoptosis of glomerular mesangial cells, leading to increased levels of reactive oxygen species and PCT. This constitutes the cytotoxic effect of contrast media, given the current understanding. Thus, the interaction of inflammatory responses, oxidative stress, and hypoxia of medullary medulla mutually promote the renal toxicity that is due to contrast media, and contribute to the pathogenesis of CIN.

The present study also found that a history of AMI was an independent risk factor for CIN after PCI. Silvain et al. [19] determined that as many as 18.3% of patients with AMI developed CIN after PCI. Kaltsas et al. [20] confirmed the high rate of CIN in patients with AMI, and that AMI severely affected the clinical prognosis of CIN. In addition to the toxicity of contrast media, some reports revealed that the high risk of CIN in patients with AMI may be due to renal hypoperfusion and neurohormonal activation [21].

All the patients in the present study were fully hydrated before and after the PCI, and administrated a small dosage of furosemide. Yet, it was found that CRP and PCT were associated with CIN occurring after PCI. Putzu et al. [22] conducted a meta-analysis that verified the efficacy and safety of furosemide with matched hydration for preventing CIN in patients undergoing PCI. In addition, Gu et al. [23]found that sufficient hydration and a small dose of furosemide after the procedure can significantly reduce the rate of CIN. In the present study, adequate hydration and low-dose furosemide was administered to reduce the incidence of CIN and improve the prognosis after PCI.

Some limitations should be noted in this study. Only preoperative baseline levels of CRP and PCT were tested, and changes in these inflammatory biomarkers during the study were not monitored. Serum creatinine was measured only before and 48 h after PCI, which potentially underestimated the incidence of CIN. Moreover, the specificity of the combination of these markers in predicting CIN was only 54.6%, and the relatively small sample size may have weakened the statistical power of the results. Therefore, we will conduct a large-sample, multi-center, randomized controlled trial to determine further the value of hs-CRP and PCT for predicting the risk of CIN.

## Conclusions

The present study demonstrates that, elevated preoperative levels of the inflammatory biomarkers hs-CRP and PCT are risk factors for the onset of CIN after PCI. The combination of elevated hs-CRP and PCT can better predict a high risk of CIN compared with either of these alone. This study may provide a simple and easy indicator to predict the risk of CIN. Therefore, a large-sample, multi-center, randomized controlled trial should be launched to verify the value of hs-CRP and PCT in predicting the risk of CIN.

## Data Availability

The datasets used and/or analysed during the current study are available from the corresponding author on reasonable request.

## Abbreviations

AUC: Area under the receiver operating characteristic curve
CI: Confidence interval
CIN: Contrast-induced nephropathy
CRP: C-reactive protein
hs-CRP: High-sensitivity C-reactive protein
PCI: Percutaneous coronary intervention
PCT: Procalcitonin
ROC: Receiver operating characteristic
